# Conventional versus miniaturized cardiopulmonary bypass: A systematic review and meta-analysis

**DOI:** 10.1016/j.xjon.2021.09.037

**Published:** 2021-10-01

**Authors:** Timothy Cheng, Rajas Barve, Yeu Wah Michael Cheng, Andrew Ravendren, Amna Ahmed, Steven Toh, Christopher J. Goulden, Amer Harky

**Affiliations:** a Faculty of Medicine, Imperial College School of Medicine, Imperial College London, London, United Kingdom; b University of Liverpool School of Medicine, Liverpool, United Kingdom; c Department of Cardiothoracic Surgery, Liverpool Heart and Chest, Liverpool, United Kingdom

**Keywords:** minimal extracorporeal circulation, cardiopulmonary bypass, cardiac surgery, coronary-artery bypass grafting, meta-analysis, AKI, acute kidney injury, CABG, coronary artery bypass graft, CECC, conventional extracorporeal circulation, CI, confidence interval, CPB, cardiopulmonary bypass, FFP, fresh-frozen plasma, ICU, intensive care unit, IL-6, interleukin-6, IL-8, interleukin-8, MECC, miniaturized extracorporeal circulation, MI, myocardial infarction, OR, odds ratio, POAF, postoperative atrial fibrillation, RBC, red blood cells, RCT, randomized control trial

## Abstract

**Objective:**

A meta-analysis of randomized controlled trials was performed to compare the effects of miniaturized extracorporeal circulation (MECC) and conventional extracorporeal circulation (CECC) on morbidity and mortality rates after cardiac surgery.

**Methods:**

A comprehensive literature search was conducted using Ovid, PubMed, Medline, EMBASE, and the Cochrane databases. Randomized controlled trials from the year 2000 with n > 40 patients were considered. Key search terms included variations of “mini,” “cardiopulmonary,” “bypass,” “extracorporeal,” “perfusion,” and “circuit.” Studies were assessed for bias using the Cochrane Risk of Bias tool. The primary outcomes were postoperative mortality and stroke. Secondary outcomes included arrhythmia, myocardial infarction, renal failure, blood loss, and a composite outcome comprised of mortality, stroke, myocardial infarction and renal failure. Duration of intensive care unit, and hospital stay was also recorded.

**Results:**

The 42 studies eligible for this study included a total of 2154 patients who underwent CECC and 2196 patients who underwent MECC. There were no significant differences in any preoperative or demographic characteristics. Compared with CECC, MECC did not reduce the incidence of mortality, stroke, myocardial infarction, and renal failure but did significantly decrease the composite of these outcomes (odds ratio, 0.64; 95% confidence interval [CI], 0.50-0.81; *P* = .0002). MECC was also associated with reductions in arrhythmia (odds ratio, 0.67; 95% CI, 0.54-0.83; *P* = .0003), blood loss (mean difference [MD], –96.37 mL; 95% CI, –152.70 to –40.05 mL; *P* = .0008), hospital stay (MD, –0.70 days; 95% CI, –1.21 to –0.20 days; *P* = .006), and intensive care unit stay (MD, –2.27 hours; 95% CI, –3.03 to –1.50 hours; *P* < .001).

**Conclusions:**

MECC demonstrates clinical benefits compared with CECC. Further studies are required to perform a cost–utility analysis and to assess the long-term outcomes of MECC. These should use standardized definitions of endpoints such as mortality and renal failure to reduce inconsistency in outcome reporting.

## Graphical abstract

Patients included in this study with their respective outcomes.

**Figure:**
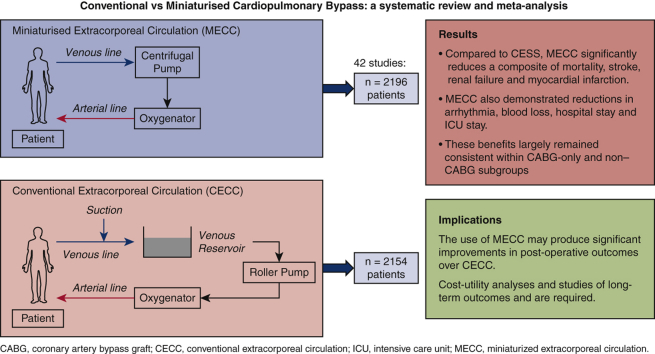

**Figure:**
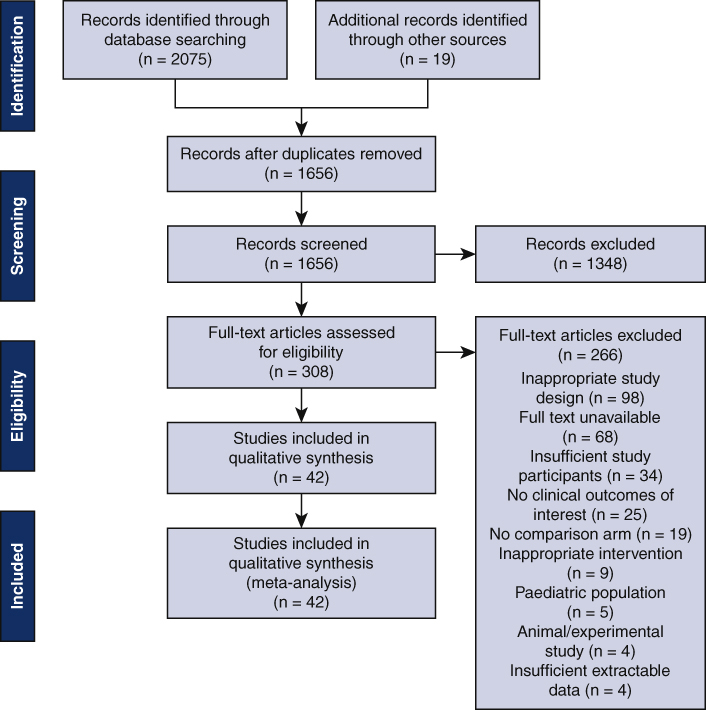
PRISMA chart showing literature search method and the results.

**Central Message**

MECC demonstrates clear postoperative benefits over CECC, reducing a composite of mortality, stroke, renal failure, and myocardial infarction.

**Perspective**

MECC significantly reduces a composite of mortality, stroke, renal failure, and myocardial infarction compared with CECC after cardiac surgery. It also demonstrates reductions in blood loss, transfusion requirements, and arrhythmia. Further analysis should determine the economic viability of MECC and compare long-term outcomes in patients undergoing MECC and CECC.

See Commentaries on pages 442 and 444.

Cardiopulmonary bypass (CPB) with cardioplegic arrest is the gold standard perfusion technique in cardiac surgery.^1^ Its use produces a systemic inflammatory response that is implicated in several severe postoperative complications, including cerebral dysfunction, myocardial depression, and hemodynamic collapse.^2^, ^3^, ^4^, ^5^, ^6^ A leading cause of this is the contact of blood components with artificial surfaces in the CPB circuit, although the development of ischemia–reperfusion injury and the presence of endotoxemia have also been implicated.^7^

Miniaturized extracorporeal circulation (MECC) was developed as a more biocompatible alternative to conventional extracorporeal circulation (CECC).^8^ This consists of a small, closed, heparin-coated circuit in which venous blood is returned to a membrane diffusion oxygenator via active drainage. This is achieved with a rotary blood pump instead of a roller pump, reducing mechanical trauma. Other advantages include the use of a cell saver to separate shed blood from the systemic circulation, low priming volumes to minimize hemodilution, and the avoidance of cardiotomy suction devices or a venous reservoir to prevent air–blood contact.^9^ These alterations could significantly attenuate the inflammatory response to CPB and therefore prevent its associated complications.

Currently, only 10% to 20% of cardiothoracic surgical units in the United Kingdom are using MECC.^10^^,^^11^ Although multiple randomized control trials (RCTs) have suggested the potential beneficial effects of MECC over CECC, previous meta-analyses disagree on its exact clinical benefits. These findings may be influenced by increasing user familiarity with MECC, the recent refinements to its technology, and the lack of large comprehensive studies comparing them, which led to the publication of the first MECC guidelines in 2017.^12^^,^^13^ Given these recent developments, this study aims to provide an updated systematic review and meta-analysis on the comprehensive outcomes of MECC, compared with CECC, in both coronary artery bypass graft (CABG) and non-CABG surgeries by including new large-scale RCTs.

## Methods

### Literature Search Strategy

A comprehensive literature search was performed using Ovid, PubMed, Medline, EMBASE, and the Cochrane Library to identify relevant articles in accordance with Preferred Reporting Items for Systematic Reviews and Meta-Analysis in October 2020. The search was confined to randomized controlled trials from 2000 to 2020. Key search terms included variations of “mini,” “cardiopulmonary,” “bypass,” “extracorporeal,” “perfusion,” and “circuit.” Search terms were combined using proximity connectors and Boolean operators to enable precision and sensitivity. Following the search, titles, and abstracts were extracted into Covidence. The full texts of the studies fulfilling the preliminary criteria were extracted, which were then read to identify studies that fulfilled the full criteria. At every stage, each paper was screened by 2 of the authors according to the inclusion and exclusion criteria below. Any discrepancies were settled by two independent members of the team (T.C. and M.C.). The final list of papers was extracted for bias assessment and data extraction.

### Inclusion and Exclusion Criteria

Studies were confined to those in the English language. Inclusion criteria included direct comparative studies of MECC and CECC, RCTs, human studies, studies with n > 40 people, and studies reporting more than 2 primary or secondary outcomes. Exclusion criteria included studies on pediatric cohorts, experimental studies, and studies with no clinical outcomes of relevance. Cohort studies, narrative reviews, and editorials were also excluded.

## Methodologic Quality Assessment of Included Studies

Qualitative analysis of the studies matching inclusion and exclusion criteria was performed to assess for bias using the Cochrane Risk of Bias tool. The scale assesses the study in each of the following 5 domains: randomization process, deviations from intended interventions, missing outcome data, measurement of the outcomes, and selection of the reported result. Each domain was rated as low risk, some concern or high risk, and the scores were combined to give an overall bias judgment (Table E1). Each paper was reviewed by 2 investigators, and any discrepancies were resolved by 2other investigators (A.R. and R.B.).

### Data Extraction and Measured Outcomes

The primary outcomes for this paper were postoperative mortality and stroke. Secondary outcomes included arrhythmia, myocardial infarction (MI), renal failure, mean blood loss, and a composite outcome of mortality, stroke, MI, and renal failure, which was calculated from the extracted data. Renal failure was defined in most studies using the Acute Kidney Injury Network stage 1 criteria but in some were referred to without explicit definition. Other outcomes measured were duration of hospital stay, intensive care unit (ICU) stay, transfusion volumes of red blood cells (RBCs), fresh-frozen plasma (FFP) and platelets, neurologic events (delirium and focal neurologic deficits), and serum interleukin-6 (IL-6) and interleukin-8 (IL-8) levels. Where available, these outcomes, as well as data regarding demographics and perioperative characteristics, were extracted from each study. This was done by 2 independent investigators, with any discrepancies resolved by the senior author.

Studies were also split into those that reported outcomes from CABG procedures and those that did not (n = 2851 and n = 1499, respectively). For each outcome, statistical comparisons were made within these subgroups in addition to the entire cohort.

### Statistical Analysis

This meta-analysis was performed in-line with recommendations from the Preferred Reporting Items for Systematic Reviews and Meta-Analysis guidelines,^14^ with all statistical analyses performed using Review Manager V.5.2.1 (Cochrane Collaboration, Oxford, United Kingdom). Random-effects models were used with inverse variance analysis or Mantel-Haenszel tests; these were chosen to account for the wide variability in sample sizes and statistical dispersion of studies. This allowed for study level means to be combined and summarized and for the standard deviations to be computed taking sample size into consideration as a proportion. Demographics and operative characteristics were compared using statistical means and *t* test. Clinical outcomes were assessed using standard meta-analysis techniques, with odds ratios (OR) or weighted mean differences (MDs) used as summary statistics to assess clinical outcomes from raw data extracted from each included study. χ^2^ tests were used to assess heterogeneity, with the Tau^2^ being calculated to describe variance between studies, and I^2^ statistic used to represent the approximate proportion of total variability due to the heterogeneity as opposed to sampling error. A Z test for overall effect was used to examine the statistical significance of the pooled estimates. Two-tailed tests were conducted. 95% confidence intervals (CIs) were used.

A sensitivity analysis was conducted by removing studies with large patient cohorts (n ≥ 200), to determine whether they biased the reported outcome. To assess for publication bias, funnel plots were constructed for primary and secondary outcomes.

## Results

### Included Studies (Study Selection)

A total of 1656 nonduplicated papers were yielded through the initial search and additional records identified through other sources. After reviewing abstract and title, 1348 records were excluded based on inclusion and exclusion criteria. The remaining records were further evaluated for eligibility through full text screening, yielding 42 studies to be included in the qualitative and quantitative analysis of this meta-analysis, as seen in central image.^15^, ^16^, ^17^, ^18^, ^19^, ^20^, ^21^, ^22^, ^23^, ^24^, ^25^, ^26^, ^27^, ^28^, ^29^, ^30^, ^31^, ^32^, ^33^, ^34^, ^35^, ^36^, ^37^, ^38^, ^39^, ^40^, ^41^, ^42^, ^43^, ^44^, ^45^, ^46^, ^47^, ^48^, ^49^, ^50^, ^51^, ^52^, ^53^, ^54^, ^55^, ^56^

### Study Characteristics

The characteristics of the included studies are described in Table 1. A total of 30 of the 42 studies looked at CABG, whereas the remaining studies either looked at CABG in addition to other procedures or only looked at non-CABG procedures, which mainly entailed heart valve surgeries. The most common MECC device, used by 18 of the included studies, was the Maquet CPB system, and the most common coating agent used for tubing was heparin.

**Table 1:** Study characteristics

Author	Year	Type of surgery	Number of participants (MECC/CECC)	MECC device: manufacturer, location	Circuit coating, MECC	Circuit coating, CECC	Priming volume (MECC/CECC), mL
Abdel-Rahman et al^15^	2005	CABG	101/103	CorX system, Jostra AG, Hirrlingen, Germany	Uncoated	Uncoated	500/1750
Anastasiadis et al^16^	2010	CABG	50/49	Maquet Jostra Cardiopulmonary, Hirrlingen, Germany	Heparin	Uncoated	500/1500
Anastasiadis et al^17^	2017	CABG	75/75	Maquet Jostra Cardiopulmonary, Hirrlingen, Germany	Heparin	Uncoated	500/1500
Asteriou et al^18^	2013	CABG	100/100	Maquet Jostra Cardiopulmonary, Hirrlingen, Germany	Heparin	Uncoated	500/1500
Basciani et al^19^	2016	AVR	24/24	Maquet Jostra Cardiopulmonary, Rastatt, Germany	Uncoated	Uncoated	600/1200
Bauer et al^20^	2010	CABG	18/22	Maquet Jostra Cardiopulmonary, Hirrlingen, Germany	Heparin	Uncoated	860/1500
Baumbach et al^21^	2016	Valve surgery	101/99	Maquet Jostra Cardiopulmonary, Hirrlingen, Germany	Heparin	Heparin	225/1337
Beghi et al^22^	2006	CABG	30/30	Maquet Jostra Cardiopulmonary, Hirrlingen, Germany	Heparin	Uncoated	450/1500
Camboni et al^23^	2009	CABG	52/40	Maquet Jostra Cardiopulmonary, Hirrlingen, Germany; PRECiSE, Medos Medizintechnik AG, Stolberg, Germany; Medtronic Resting Heart System, Dusseldorf, Germany	Heparin	Uncoated	500/1200
Castiglioni et al^24^	2007	AVR	17/23	Maquet Jostra Cardiopulmonary, Rastatt, Germany	Phosphorylcholine	Phosphorylcholine	500/1500
Castiglioni et al^25^	2009	AVR	60/60	Maquet Jostra Cardiopulmonary, Rastatt, Germany	Heparin	Phosphorylcholine	500/1600
Chew et al^26^	2015	CABG	34/33	Extra Corporeal Circuit Optimized (Sorin Group, Mirandola, Italy)	Phosphorylcholine	Phosphorylcholine	850/1350
Deininger et al^27^	2016	CABG	36/39	Maquet Jostra Cardiopulmonary, Rastatt, Germany	Heparin	Uncoated	<600/750
El-Essawi et al^28^	2011	CABG; AVR; CABG + AVR	252/248	ROCsafeRXTM MPC, Terumo Cardiovascular Systems, Ann Arbor, Mich	X-coating	Uncoated	150/1500
Elçi et al^29^	2019	CABG	31/27	Maquet Jostra Cardiopulmonary, Hirrlingen, Germany	Heparin	Uncoated	800/1650
Farag et al^30^	2016	CABG	20/20	Maquet Jostra Cardiopulmonary, Rastatt, Germany	Bioline	N/A	750/1100
Gunaydin et al^31^	2009	CABG	20/20	ROCsafeRXTM MPC, Terumo Cardiovascular Systems, Ann Arbor, Mich	PMEA	Uncoated	800/1360
Gygax et al^32^	2018	AVR	24/26	Maquet Jostra Cardiopulmonary, Hirrlingen, Germany	Uncoated	Uncoated	600/1500
Halfwerk et al^33^	2019	Aortic valve surgery	63/62	Maquet Jostra Cardiopulmonary, Hirrlingen, Germany	Bioline	Bioline	800/1500
Haneya et al^34^	2012	CABG	50/50	Maquet Jostra Cardiopulmonary, Hirrlingen, Germany	Heparin	Heparin	500/500
Huybregts et al^35^	2007	CABG	25/24	Synergy Mini-bypass system (Cobe), Rastatt, Germany	Phosphorylcholine	Phosphorylcholine	393/1330
Kiaii et al^36^	2012	CABG	20/20	Medtronic Resting Heart System, Dusseldorf, Germany	Heparin	Uncoated	750/1000
Kiessling et al^37^	2018	CABG	24/26	Maquet Jostra Cardiopulmonary, Hirrlingen, Germany	Bioline	Softline coating	600/1290
Kofidis et al^38^	2008	CABG	50/30	Maquet Jostra Cardiopulmonary, Hirrlingen, Germany	Heparin coated	N/A	500/NA
Kolackova et al^39^	2012	CABG	22/22	Minisystem Synergy, Sorin Group, Mirandola, Italy	Phosphorylcholine	N/A	1100/1600
Kutschka et al^40^	2009	Aortic valve surgery (+/– CABG); aortic root surgery	85/85	ROCsafeRXTM MPC, Terumo Cardiovascular Systems, Ann Arbor, Mich	X-coating	X-coating	<400/1000
Liu et al^41^	2012	CABG	20/20	Maquet Jostra Cardiopulmonary, Hirrlingen, Germany	Heparin	Uncoated	1000/1500
Modrau et al^42^	2020	CABG	30/30	Affinity, Medtronic International, Tolochenaz, Switzerland	Biocompatible	Biocompatible	400/1400
Nasso et al^43^	2011	CABG; valve surgery; combined	77/73	EVADO system: ADMIRAL (Eurosets, Medolla, Italy); HARMONY (Haemonetics, Braintree, Mass)	Heparin	Heparin	750/1000
Ng et al^44^	2015	CABG	39/39	Phisio, Sorin Group, Mirandola, Italy	Phosphorylcholine	Phosphorylcholine	850/1350
Ohata et al^45^	2008	CABG	34/64	Capiox, Terumo, Tokyo, Japan	PMEA	PMEA	750/1600
Remadi et al^46^	2004	AVR	50/50	Maquet Jostra Cardiopulmonary, Hirrlingen, Germany	Heparin	Uncoated	450/1700
Remadi et al^47^	2006	AVR	200/200	Bioline-Jostra, Gretz, France	N/A	N/A	450/1700
Rimpiläinen et al^48^	2011	AVR	20/20	Maquet Jostra Cardiopulmonary, Rastatt, Germany	PMEA	Phosphorylcholine	N/A
Rosato et al^49^	2012	CABG	18/21	Maquet Jostra Cardiopulmonary, Hirrlingen, Germany	Carmeda	Uncoated	900/1300
Sakwa et al^50^	2009	CABG	102/97	Medtronic Resting Heart System, Dusseldorf, Germany	Heparin	N/A	900/1850
Schoenebeck et al^51^	2010	CABG	80/40	Maquet Jostra Cardiopulmonary, Hirrlingen, Germany	Heparin	Uncoated	760/1600
Schöttler et al^52^	2008	CABG	30/30	Maquet Jostra Cardiopulmonary, Hirrlingen, Germany	N/A	N/A	900/1700
Skrabal et al^53^	2007	CABG	30/30	Maquet Jostra Cardiopulmonary, Hirrlingen, Germany	Heparin	Heparin	500/1500
Svitek et al^54^	2009	CABG	26/28	Minisystem Synergy, Sorin Group, Mirandola, Italy	Phosphorylcholine	Heparin	600/1100
Van Boven et al^55^	2013	CABG	20/20	Maquet Jostra Cardiopulmonary, Hirrlingen, Germany	Bioline	N/A	500/1000
Yuhe et al^56^	2020	CABG	36/35	Phisio, Sorin Group, Mirandola, Italy	Phosphorylcholine	Phosphorylcholine	800/1300

*MECC*, Miniaturized extracorporeal circulation; *CECC*, conventional extracorporeal circulation; *CABG*, coronary artery bypass graft; *AVR*, aortic valve replacement; *N/A*, not available; *PMEA*, polymethoxyethylacrylate.

### Demographics and Operative Characteristics

The 42 studies eligible for this study included a total of 2154 patients who underwent CECC and 2196 patients who underwent MECC. Detailed description of the preoperative and operative characteristics of each cohort is given in Table 2. The age and sex ratios were similar between both cohorts. Hypertension was the most prevalent preoperative comorbidity among this population and the incidence of hypertension between the 2 cohorts were similar. CBP times (CECC: 95 ± 24 vs MECC: 94 ± 25, *P* = .15) and aortic cross clamp times (CECC: 57 ± 15 vs MECC: 58 ± 18, *P* = .61) were not significantly different between both procedures. The average priming volume was significantly reduced in MECC compared with CECC (649 ± 171 vs 1424 ± 350, *P* < .001).

**Table 2:** Preoperative characteristics and operative factors

	MECC (n = 2196)	CECC (n = 2154)
Preoperative characteristics		
Age, y, mean ± SD	66 ± 8	66 ± 8
Male (%)	1394/1878 (74%)	1368/1854 (73%)
BMI, mean ± SD	26 ± 4	26 ± 5
Logistic EuroSCORE, mean ± SD	4 ± 2	4 ± 2
LVEF, mean ± SD	56 ± 10	56 ± 10
Stroke, n (%)	18/625 (3%)	18/604 (3%)
Diabetes mellitus, n (%)	387/1521 (25%)	401/1501 (27%)
Hypertension, n (%)	931/1437 (65%)	891/1415 (63%)
Ischaemic heart disease, n (%)	237/1130 (21%)	230/1116 (21%)
COPD, n (%)	117/1296 (9%)	97/1277 (8%)
Atrial fibrillation, n (%)	26/243 (11%)	25/227 (11%)
Renal Insufficiency, n (%)	102/831 (12%)	99/814 (12%)
Operative factors		
CPB time, min, mean ± SD	94 ± 25	95 ± 24
Crossclamp time, min, mean ± SD	58 ± 18	58 ± 17
Priming volume, mL, mean ± SD	649 ± 171	1424 ± 350
CABG only (n = 2851)		
CPB time, min, mean ± SD	94 ± 24	96 ± 24
Crossclamp time, min, mean ± SD	57 ± 16	57 ± 15
Priming volume, mL, mean ± SD	665 ± 173	1418 ± 343
Non-CABG only (n = 1499)		
CPB time, min, mean ± SD	91 ± 26	91 ± 27
Crossclamp time, min, mean ± SD	63 ± 24	63 ± 24
Priming volume, mL, mean ± SD	592 ± 162	1449 ± 375

*MECC*, Miniaturized extracorporeal circulation; *CECC*, conventional extracorporeal circulation; *SD*, standard deviation; *BMI*, body mass index; *EuroSCORE*, European System for Cardiac Operative Risk Evaluation; *LVEF*, left ventricular ejection fraction; *COPD*, chronic obstructive pulmonary disease; *CPB*, cardiopulmonary bypass; *CABG*, coronary artery bypass graft.

### Assessment of Publication Bias

**Figure E1:**
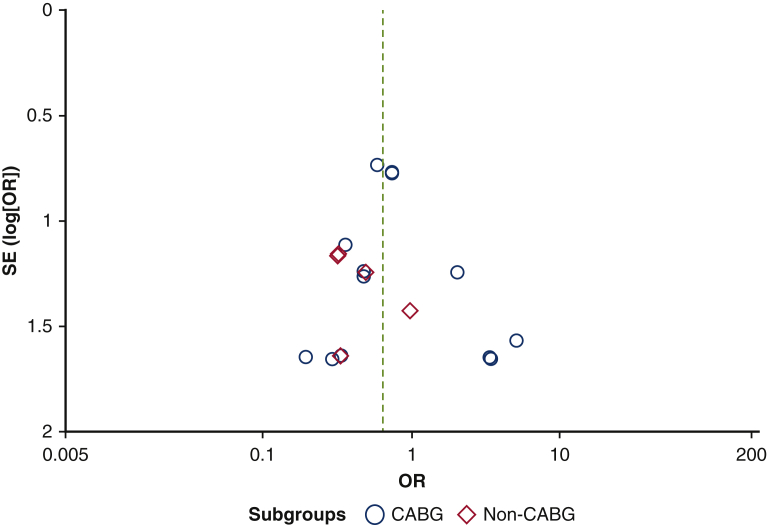
Funnel plot assessing mortality for publication bias comparing CECC and MECC. *SE*, Standard error; *OR*, odds ratio; *CABG*, coronary artery bypass graft.

**Figure E2:**
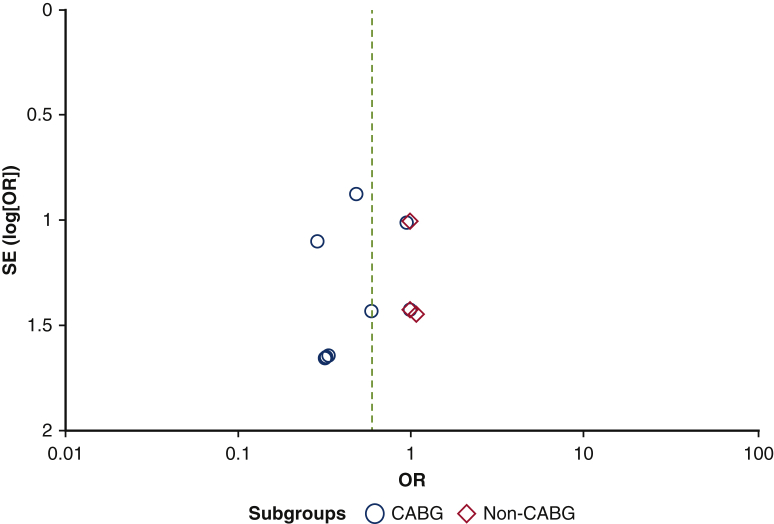
Funnel plot assessing stroke outcome for publication bias comparing CECC and MECC. *SE*, Standard error; *OR*, odds ratio; *CABG*, coronary artery bypass graft.

**Figure E3:**
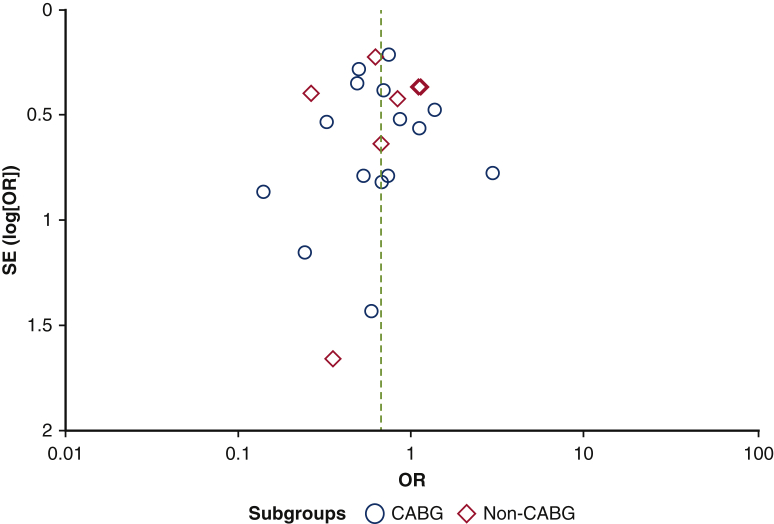
Funnel plot assessing arrythmia for publication bias comparing CECC and MECC. *SE*, Standard error; *OR*, odds ratio; *CABG*, coronary artery bypass graft.

**Figure E4:**
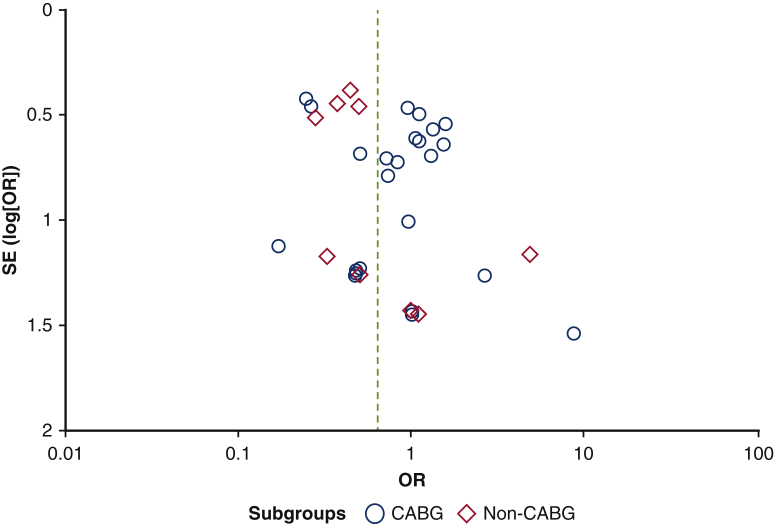
Funnel plot assessing composite outcome for publication bias comparing CECC and MECC. *SE*, Standard error; *OR*, odds ratio; *CABG,* coronary artery bypass graft.

**Figure E5:**
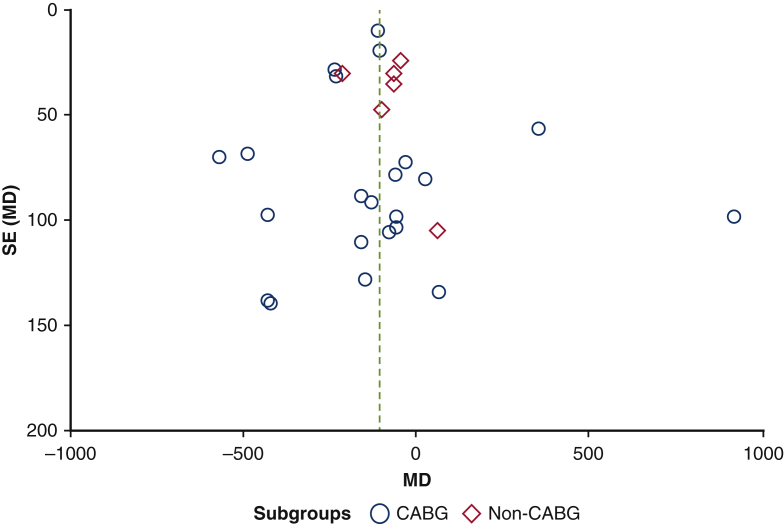
Funnel plot assessing mean blood loss outcome for publication bias comparing CECC and MECC. *SE,* Standard error; *MD*, weighted mean difference; *CABG*, coronary artery bypass graft.

**Figure E6:**
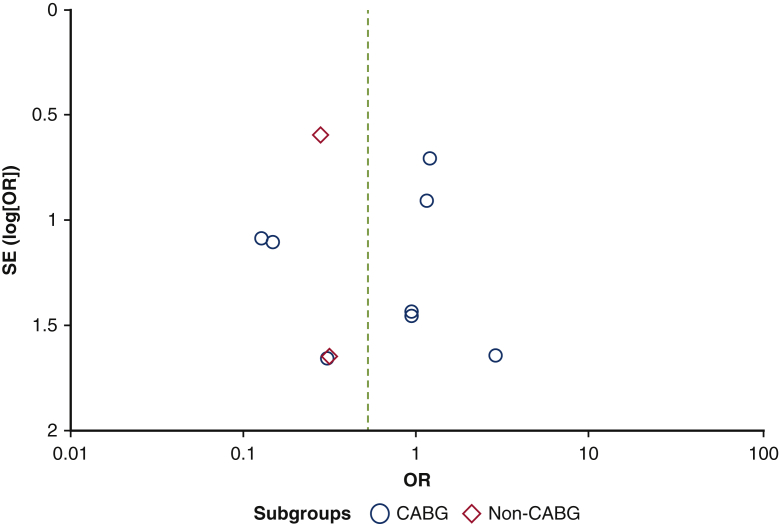
Funnel plot assessing myocardial infarction outcome for publication bias comparing CECC and MECC. *SE,* Standard error; *OR*, odds ratio; *CABG*, coronary artery bypass graft.

**Figure E7:**
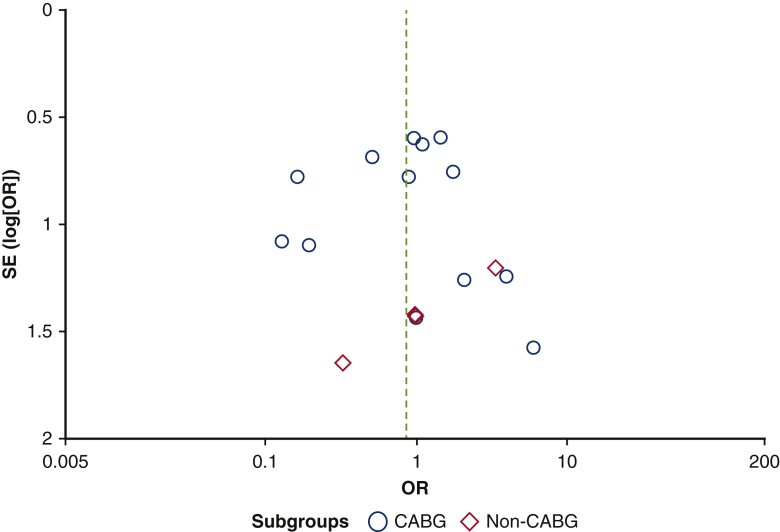
Funnel plot assessing renal failure outcome for publication bias comparing CECC and MECC. *SE*, Standard error; *OR,* odds ratio; *CABG,* coronary artery bypass graft.

Funnel plots of primary and secondary outcomes yielded symmetrical shapes, indicating minimal publication bias. Most studies included within this meta-analysis had small sample sizes (n < 100) and there was no evidence that statistically insignificant results were excluded from these studies. All funnel plots used to assess publication bias are displayed in Figure E1, Figure E2, Figure E3, Figure E4, Figure E5, Figure E6, Figure E7.

### Postoperative Data

#### Primary outcomes

**Figure 1:**
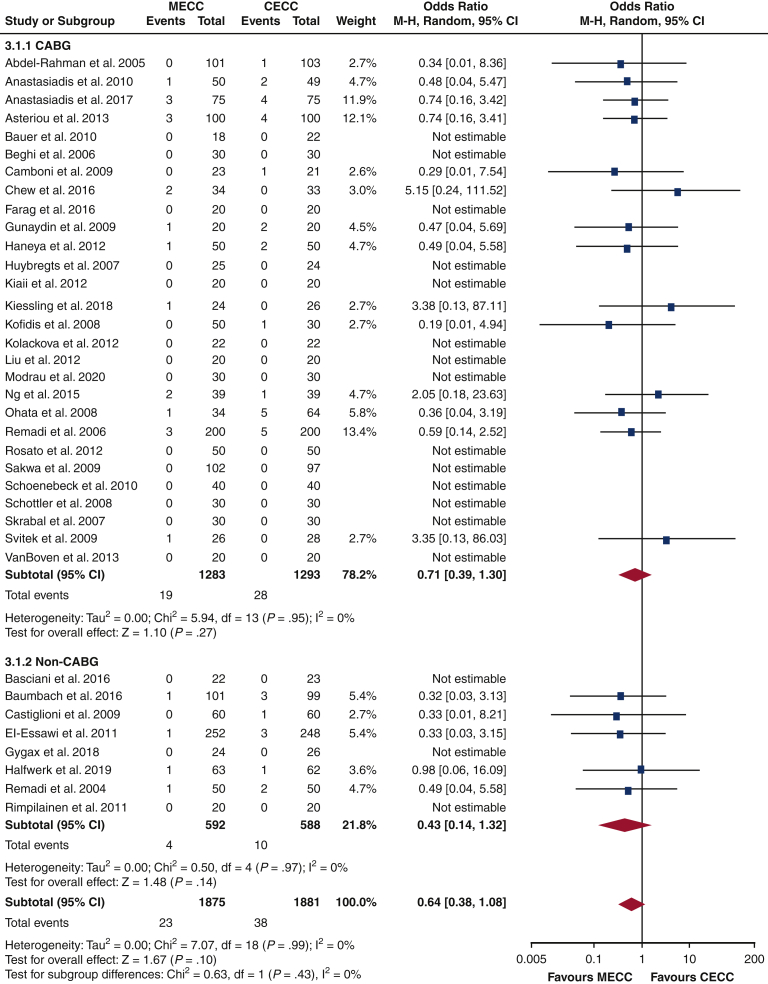
Forest plot for mortality rate in comparing CECC and MECC. *MECC*, Miniaturized extracorporeal circulation; *CECC*, conventional extracorporeal circulation; *M-H*, Mantel-Haenszel; *CI*, confidence interval; *CABG*, coronary artery bypass graft; *df*, degrees of freedom.

The postoperative data for the entire cohort are presented in Table 3, and the postoperative data for CABG and non-CABG subgroups are shown in Table 4. There was no significant difference in mortality in the MECC cohort compared with the CECC cohort (OR, 0.64; 95% CI, 0.38-1.08; test for overall effect: *P* = .10, Figure 1). This was also the case within CABG and non-CABG cohorts (OR, 0.71; 95% CI, 0.39-1.30; *P* = .27; OR, 0.43; 95% CI, 0.14-1.32; *P* = .14, respectively). Accordingly, the test for subgroup differences gave a nonsignificant result (*P* = .43).

**Table 3:** Postoperative data for entire cohort

	MECC (n = 2196)	CECC (n = 2154)
Mortality	23/1875 (1%)	38/1881 (2%)
Stroke	13/1332 (1%)	25/1338 (2%)
Renal failure	50/1046 (5%)	63/1079 (6%)
Myocardial infarction	21/992 (2%)	39/975 (4%)
Composite outcome: (mortality, stroke, renal failure, myocardial infarction)	159/2116 (8%)	237/2124 (11%)
Arrhythmia	319/1372 (23%)	422/1355 (31%)
Mean blood loss	612 ± 311	706 ± 407
Hospital stay, d	9 ± 4	10 ± 5
ICU stay, h	32 ± 18	40 ± 37
Reoperation	30/1243 (2%)	55/1228 (4%)
Sternal wound infection	12/482 (2%)	17/514 (3%)
Neurologic events	56/901 (6%)	82/933 (9%)
Transfusion volume RBC	515 ± 492	772 ± 589
Transfusion volume FFP	261 ± 285	473 ± 467
Transfusion volume platelets	9 ± 9	18 ± 20
IL-6, ng/L	167 ± 90	179 ± 89
IL-8, ng/L	19 ± 11	25 ± 16

*MECC*, Miniaturized extracorporeal circulation; *CECC*, conventional extracorporeal circulation; *ICU*, intensive care unit; *RBC*, red blood cells; *FFP*, fresh-frozen plasma; *IL-6*, interleukin-6; *IL-8*, interleukin-8.

**Table 4:** Postoperative data for CABG and non-CABG subgroups

	MECC (n = 2196)	CECC (n = 2154)
CABG-only (n = 2851)		
Mortality	19/1283 (1%)	28/1293 (2%)
Stroke	9/793 (1%)	21/797 (3%)
Renal failure	45/742 (6%)	59/773 (8%)
Myocardial infarction	17/583 (3%)	25/564 (4%)
Composite outcome: (mortality, stroke, renal failure, myocardial infarction)	119/1216 (10%)	156/1224 (13%)
Arrhythmia	191/830 (23%)	254/812 (31%)
Mean blood loss	666 ± 355	759 ± 454
Hospital stay, d	10 ± 4	11 ± 5
ICU stay, h	34 ± 18	45 ± 39
Reoperation	19/691 (3%)	19/675 (3%)
Sternal wound infection	12/376 (3%)	17/405 (4%)
Neurologic events	34/563 (6%)	40/596 (7%)
Transfusion volume RBC	591 ± 532	883 ± 626
Transfusion volume FFP	305 ± 263	627 ± 483
Transfusion volume platelets	6 ± 3	13 ± 6
IL-6, ng/L	151 ± 94	154 ± 96
IL-8, ng/L	17 ± 11	24 ± 18
Non-CABG only (n = 1499)		
Mortality	4/592 (1%)	10/588 (2%)
Stroke	4/539 (1%)	4/541 (1%)
Renal failure	5/304 (2%)	4/306 (1%)
Myocardial infarction	4/409 (1%)	14/411 (3%)
Composite outcome: (mortality, stroke, renal failure, myocardial infarction)	40/900 (4%)	81/900 (9%)
Arrhythmia	128/542 (24%)	168/543 (31%)
Mean blood loss	442 ± 172	506 ± 228
Hospital stay, d	8 ± 3	9 ± 5
ICU stay, h	27 ± 19	30 ± 31
Reoperation	11/552 (2%)	36/553 (7%)
Sternal wound infection	0/106 (0%)	0/109 (0%)
Neurologic events	22/338 (7%)	42/337 (12%)
Transfusion volume RBC	349 ± 406	526 ± 508
Transfusion volume FFP	192 ± 319	226 ± 441
Transfusion volume platelets	12 ± 14	23 ± 35
IL-6, ng/L	252 ± 71	305 ± 50
IL-8, ng/L	24 ± 12	26 ± 11

*MECC*, Miniaturized extracorporeal circulation; *CECC*, conventional extracorporeal circulation; *CABG*, Coronary artery bypass graft; *ICU*, intensive care unit; *RBC*, red blood cells; *FFP*, fresh-frozen plasma; *IL-6*, interleukin-6; *IL-8*, interleukin-8.

**Figure 2:**
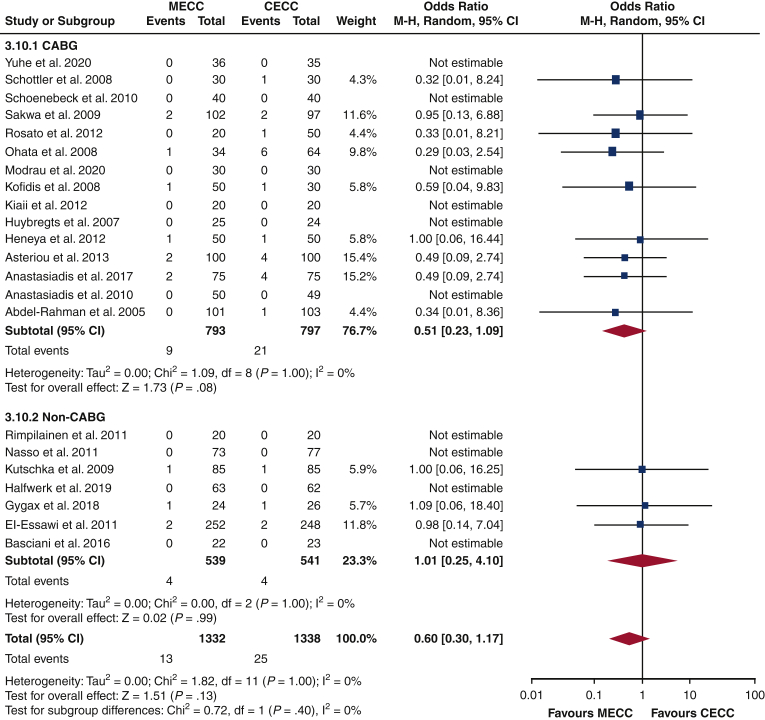
Forest plot for stroke outcomes comparing CECC and MECC. *MECC*, Miniaturized extracorporeal circulation; *CECC*, conventional extracorporeal circulation; *M-H*, Mantel-Haenszel; *CI*, confidence interval; *CABG*, coronary artery bypass graft; *df*, degrees of freedom.

**Figure 3:**
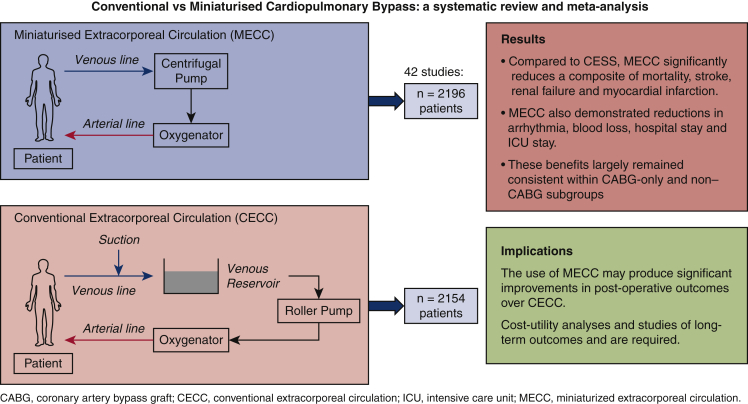
Outcomes of patients who underwent conventional extracorporeal circulation (CECC, n = 2154) versus those who underwent miniaturized extracorporeal circulation (MECC, n = 2196). No difference in mortality, stroke, myocardial infarction, and renal failure has been reported. *ICU*, Intensive care unit; *CABG*, coronary artery bypass graft;

There was no significant difference in stroke incidence between MECC and CECC cohorts (OR, 0.60; 95% CI, 0.30-1.17; *P* = .13). The analysis within CABG and non-CABG subgroups yielded similar findings (OR, 0.51; 95% CI, 0.23-1.09; *P* = .08; OR, 1.01; 95% CI, 0.25-4.10; *P* = .99, respectively). There was no significant difference the findings of the 2 subgroups (*P* = .40) (Figure 2). Other results are summarized in Figure 3.

#### Secondary outcomes

**Figure E8:**
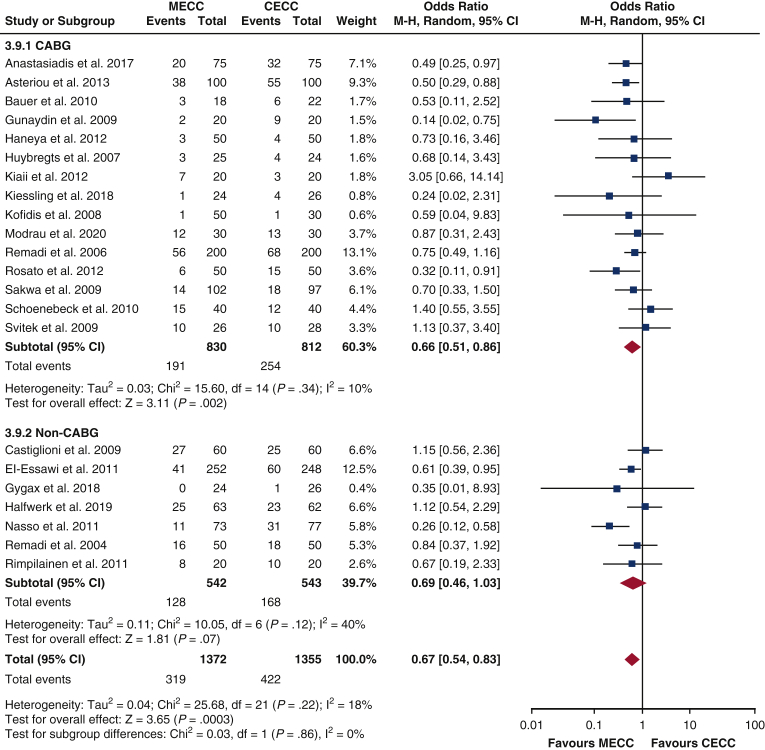
Forest plot for postoperative arrhythmia in CECC and MECC. *MECC*, Miniaturized extracorporeal circulation; *CECC,* conventional extracorporeal circulation; *M-H,* Mantel-Haenszel; *CI,* confidence interval; *CABG*, coronary artery bypass graft; *df*, degrees of freedom.

**Figure E9:**
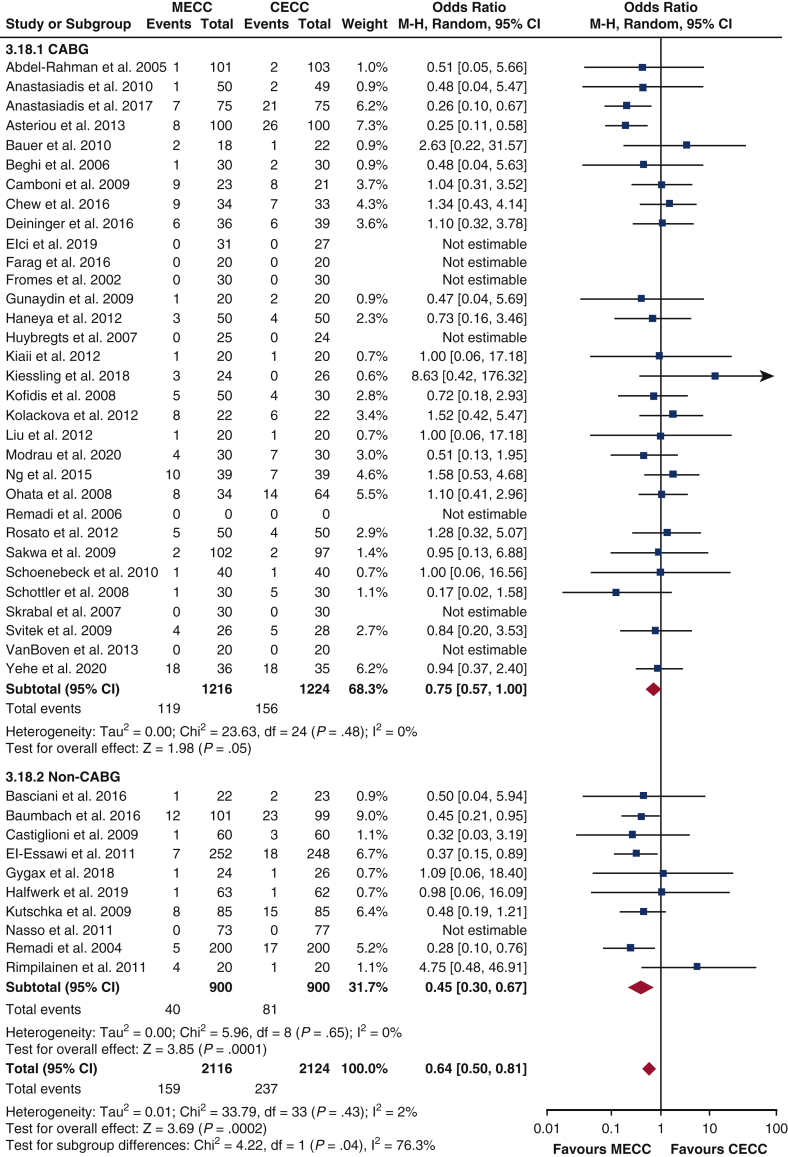
Forest plot for composite outcomes in CECC and MECC. *MECC*, Miniaturized extracorporeal circulation; *CECC*, conventional extracorporeal circulation; *M-H*, Mantel-Haenszel; *CI*, confidence interval; *CABG*, coronary artery bypass graft; *df*, degrees of freedom.

The overall incidence of arrhythmia was significantly reduced in the MECC cohort compared with the CECC cohort (OR, 0.67; 95% CI, 0.54-0.83; *P* = .0003). This reduction was also seen in the CABG-only subgroup (OR, 0.66; CI, 0.51-0.86; *P* = .002), but not in the analysis of non-CABG procedures (OR, 0.69; CI, 0.46-1.03; *P* = .07). However, the test for subgroup differences gave a non-significant result (*P* = .86) (Figure E8). A composite outcome encompassing the incidence of death, MI, stroke, and renal failure was generated. In the overall analysis, the MECC cohort demonstrated a significantly lower incidence of this outcome compared with CECC (OR, 0.64, 95% CI, 0.50-0.81; *P* = .0002). This was also seen within the non-CABG subgroup (OR, 0.45, 95% CI, 0.30-0.67; *P* = .0001). In the CABG subgroup, no difference was observed, but this verged on significance (0.75; 95% CI, 0.57-1.00; *P* = .05). There was a significant difference in the findings of the 2 subgroups (*P* = .04) (Figure E9).

**Figure E10:**
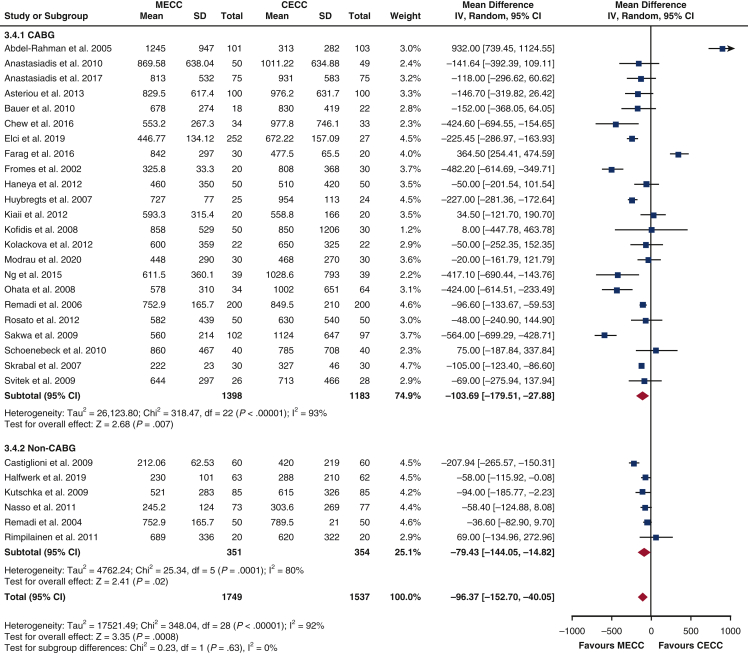
Forest plot for mean blood loss comparing CECC and MECC. *MECC*, Miniaturized extracorporeal circulation; *CECC*, conventional extracorporeal circulation; *SD*, standard deviation; *IV*, instrumental variables; *CI*, confidence interval; *CABG,* coronary artery bypass graft; *df,* degrees of freedom.

**Figure E11:**
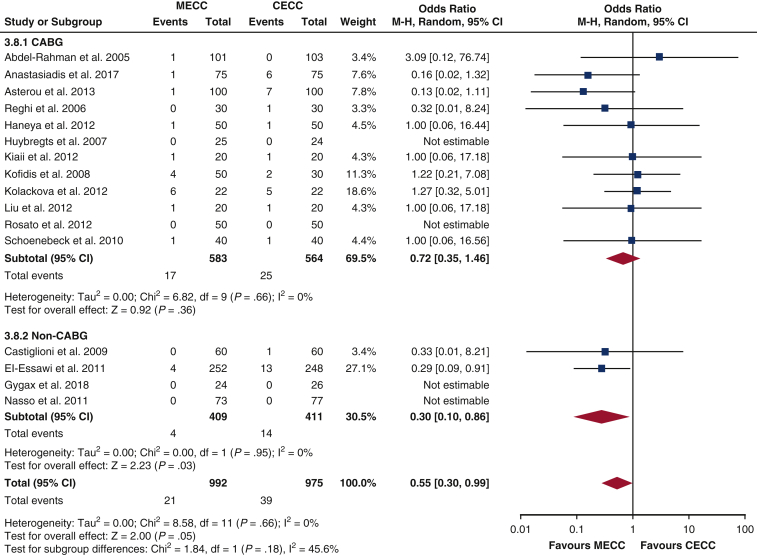
Forest plot for myocardial infarction comparing CECC and MECC. *MECC,* Miniaturized extracorporeal circulation; *CECC*, conventional extracorporeal circulation; *M-H*, Mantel-Haenszel; *CI*, confidence interval; *CABG,* coronary artery bypass graft; *df*, degrees of freedom.

**Figure E12:**
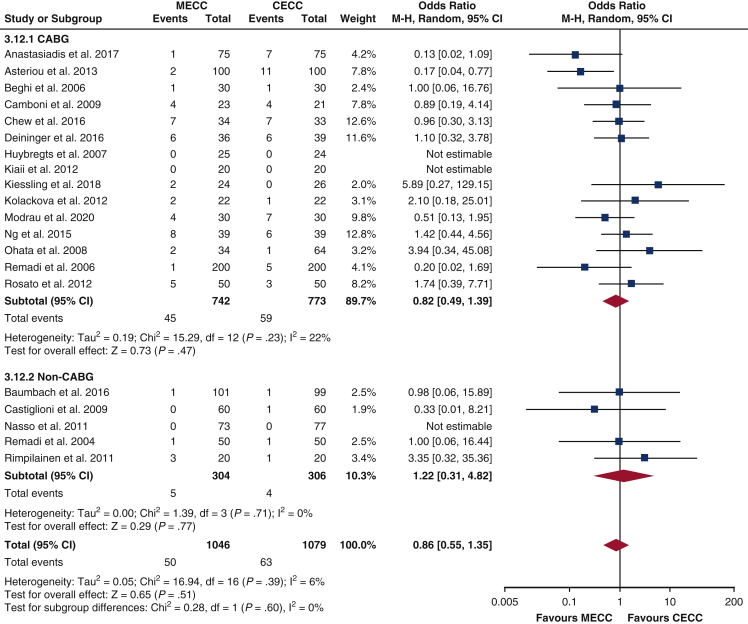
Forest plots for rate of renal failure comparing CECC and MECC. *MECC,* Miniaturized extracorporeal circulation; *CECC*, conventional extracorporeal circulation; *M-H*, Mantel-Haenszel; *CI,* confidence interval; *CABG,* coronary artery bypass graft; *df*, degrees of freedom.

Compared with CECC, MECC was associated with a reduction of mean blood loss in the overall analysis (MD, –96.37 mL; 95% CI, –152.70 to –40.05 mL; *P* = .0008) and subgroup analyses (CABG-only: MD, –103.69 mL; 95% CI, –179.51 to –27.88 mL; *P* = .007, non-CABG: MD, –79.43 mL; 95% CI, –144.05 to –14.82 mL; *P* = .02). There was no significant difference between subgroup findings (*P* = .63) (Figure E10). Overall analysis of MI incidence revealed no significant difference between MECC and CECC cohorts, however this verged on significance (OR, 0.55; 95% CI, 0.30-0.99; *P* = .05). This was also seen in subgroup analysis of patients receiving CABG only (OR, 0.72; CI, 0.35-1.46; *P* = .36). However, in non-CABG procedures, there was a reduced MI incidence with MECC (OR, 0.30; 95% CI, 0.10-0.86; *P* = .03). The findings between subgroups were not significantly different (*P* = .18) (Figure E11). There was no significant difference in postoperative renal failure incidence in the overall analysis (OR, 0.86; 95% CI, 0.55-1.35; *P* = .51). Further subgroup analysis did not reveal any significant differences either (CABG: OR, 1.82; 95% CI, 0.49-1.39; *P* = .47; non-CABG: OR, 1.22; CI, 0.31-4.82; *P* = .77). There was no significant difference between the subgroup findings (*P* = .60; Figure E12).

#### Other outcomes

MECC was associated with a significantly reduced length of hospital stay (MD, –0.70 days; 95% CI, –1.21 to –0.20 days; *P* = .006). This was also the case with ICU stay (MD, –2.27 hours; 95% CI, –3.03 to –1.50 hours; *P* < .00001). Incidence of reoperation was also significantly reduced in the MECC cohort (OR, 0.57; 95% CI, 0.36-0.90; *P* = .02). This difference was reflected in the non-CABG subgroup (OR, 0.32; 95% CI, 0.16-0.63; *P* = .001) but not the CABG-only group (OR, 0.95; 95% CI, 0.50-1.81; *P* = .88).

MECC procedures significantly reduced the transfusion volume of RBCs (MD –227.42 mL, 95% CI, –337.00 to –117.85 mL; *P* < .001). Similar reductions were observed in the volumes of transfused FFP and platelets (MD, –74.76 mL; 95% CI, –125.90 to –23.62 mL; *P* = .004; MD, –8.95 mL; 95% CI, –14.44 to –3.46 mL; *P* = .001, respectively). MECC did not affect the incidence of neurologic events in the overall analysis (OR, 0.68; 95% CI, 0.42-1.09; *P* = .11) but was beneficial in patients who did not receive CABG (OR, 0.48; 95% CI, 0.28-0.84; *P* = .009). Significantly reduced postoperative IL-6 levels were seen with MECC compared with CECC (MD, –23.61 ng/L; 95% CI, –42.13 to –5.09 ng/L; *P* = .01). Similar findings were observed with IL-8 (MD, –6.30 ng/L; 95% CI, –11.64 to –0.97 ng/L; *P* = .02).

### Sensitivity Analysis

El-Essawi and colleagues^28^ and Remadi and colleagues^47^ were removed from the analysis. Consequently, there was no longer a statistically significant difference in reoperation rates (OR, 0.76; 95% CI, 0.43-1.37, test for overall effect: *P* = .36). The incidence of MI remained statistically insignificant between groups, but the *P* value increased (OR, 0.69; 95% CI, 0.35, 1.39; *P* = .30). Overall, the sensitivity analysis did not significantly alter the findings of primary and secondary outcomes, indicating that studies with large sample sizes did not distort the results.

## Discussion

We demonstrate that MECC significantly reduces a composite incidence of postoperative mortality, stroke, renal failure and MI when compared with CECC. However, there was not sufficient evidence to show a decrease in these outcomes individually. MECC is also associated with reduced hospital and ICU stay, blood loss, transfusion requirements, reoperation rates, and IL-6 and IL-8 concentrations. These benefits largely remained consistent within CABG-only and non-CABG subgroups, with the exception of the composite outcome and reoperation rates, which did not show significant benefit in CABG-only studies.

### Primary Outcomes

#### Mortality

No significant differences were observed between MECC and CECC cohorts. This is consistent with findings from previous meta-analyses,^1^^,^^57^^,^^58^ but not with that of Kowalewski and colleagues,^59^ in which MECC was shown to reduce mortality, particularly in CABG procedures. Only some studies explicitly state the duration at which mortality is recorded, making standardization of the data difficult. Of these studies, a subgroup analysis stratified according to the different durations of reported mortality may yield a more accurate assessment. The short follow-up periods in the included studies also make assessments in long-term survival difficult.

#### Cerebrovascular and neurologic complications

Previous meta-analyses have yielded conflicting effects of MECC on neurologic outcomes. It has been hypothesized that MECC could provide a degree of protection against stroke and other neurologic events via its reduction of hemodilution, therefore preventing cerebral hypoperfusion, and its reduction of lipid micro-emboli, which can be formed by cardiotomy suction.^60^ Some meta-analyses have indeed shown significant reductions in these events postoperatively.^1^^,^^58^ However, the present article did not find any significant differences between the 2 cohorts, corroborating a more recent meta-analysis by Anastasiadis and colleagues.^61^ Similar to that study, we also argue that our analysis has the advantage of a much larger patient pool, and the inclusion of recent studies that use improved CECC circuits, for example, the use of heparin-coated surfaces, and of other modifications that reduce air entrainment. Furthermore, the degree of aortic manipulation has also been identified as a significant risk factor of neurologic injury, which was not controlled for in our analysis.^62^ Lastly, it has also been shown that the omission of a venous reservoir in MECC may potentially increase the risk of air micro-emboli formation, which can also cause cerebral ischemic injury.^1^

### Secondary Outcomes

#### Renal failure

Renal dysfunction is a common complication after cardiac procedures.^63^ The systemic inflammatory response initiated by CECC directly contributes to this by enhancing the secretion of reactive oxygen species, leading to acute tubular necrosis.^64^ Other effects of CECC such as hemodilution, microemboli formation, and erythrocyte hemolysis can also lead to renal tubular damage by other mechanisms.^65^ Postoperatively, this can manifest as an acute kidney injury (AKI) or persistence or worsening of pre-existing renal disease. MECC incorporates modifications designed to minimize these risk factors,^9^ although this present meta-analysis did not demonstrate an improvement in rates of AKI in CABG and non-CABG cohorts. This finding appears to be corroborated most by studies which concurrently did not find significant differences in postoperative inflammatory markers between the systems.^37^^,^^48^^,^^49^ In a 2016 meta-analysis, MECC demonstrated approximately one-half the odds of postoperative renal dysfunction compared with CECC; however, this analysis encompassed both instances of AKI and asymptomatic increases of creatinine of over 50% from pre-operative values.^59^ The latter events have not been included in our analysis.

#### Hematologic outcomes

Corroborating previous meta-analyses, the present study demonstrated significantly lower requirements for RBC transfusion with MECC over CECC.^61^ This is likely a result of the reduced size of the CPB circuit, which minimizes hemodilution and therefore increases hematocrit levels during surgery.^8^ Another contributor may be the lower mean postoperative blood losses and rates of surgical re-exploration for bleeding also demonstrated here. The lower requirements for platelets and FFP may instead reflect the decreased activation of coagulation cascades with MECC, an expected result of using a cell saver and avoiding blood–air contact. Minimizing peri- and postoperative transfusion rates reduces burdens on blood banks and limits the associated risks and complications.

#### Composite outcome

Although no significant differences between the cohorts were found in mortality, stroke, renal failure, and MI, MECC demonstrated a numerically lower incidence in all of these outcomes. This may partially be due to low event rates within each study and therefore an insufficient sample power to detect differences between cohorts. To increase statistical power, a composite of these outcomes was generated. MECC significantly reduced this composite outcome over CECC, supporting a clinical benefit undetected by single outcome analyses.

#### Arrhythmia

Postoperative atrial fibrillation (POAF) is a common complication of cardiac surgery with an incidence of 20% to 40% after CABG and up to 60% after valvular surgery.^66^^,^^67^ This meta-analysis found a significant reduction in arrhythmias following CABG surgery when using MECC over CECC and therefore supports previous meta-analyses.^59^^,^^61^^,^^68^ Its etiology is complex and not well understood; however, the heightened inflammatory response associated with CECC is likely to play a major role. Kourliouros and Laffey implicate C-reactive protein, IL-1, IL-6, tumor necrosis factor-alpha, and complement activation for the electrical and structural cardiac remodeling seen in the pathogenesis of POAF.^69^^,^^70^ Furthermore, Koch and colleagues^71^ report that blood transfusions following surgery can further exacerbate the inflammatory response through direct infusion of inflammatory markers. In support, this meta-analysis found a significant reduction in mean blood loss, transfusion requirements and inflammatory markers IL-6/IL-8 compared with CECC, further supporting the role of MECC in reducing the inflammatory response and incidence of POAF. This meta-analysis did not observe any significant reduction in POAF in non-CABG surgeries and is in keeping with findings from Wang and colleagues.^57^ The literature is limited in this field, and further RCTs are required to assess the specific effects of MECC in non-CABG surgeries.

#### Hospital and ICU stay

The duration of hospital and ICU stay was significantly reduced with MECC. However, the I^2^ values of 84% and 93%, respectively, suggest a high level of heterogeneity which may indicate a different true effect. There is a discrepancy in existing meta-analyses about the significance of hospital stay, which may be explained by different standards for discharge and the small numbers of papers reporting these outcomes. Reduction of stay duration may lead to reductions in cost and postoperative morbidity.

#### IL-6 and IL-8

Many included studies measured IL-6 and IL-8 to assess postoperative inflammatory responses. IL-6 is involved in interactions between neutrophils and myocytes and contributes to postoperative myocardial damage,^72^ whereas IL-8 augments the response of neutrophils and macrophages and may contribute to vascular damage.^73^ This meta-analysis identified that the sharp rise in the concentrations of IL-6 and IL-8 postsurgery is significantly dampened in patients undergoing MECC. Remadi and colleagues^74^ identified that IL-6 and IL-8 concentrations are greater in blood circulating through cardiotomy suction, which is absent in MECC.

The present article demonstrates that MECC produces significant postoperative benefits over CECC. The finding of IL-6 and IL-8 reductions with MECC affirms the notion that this technology produces a significantly attenuated inflammatory reaction. Despite these benefits, the further incorporation of MECC into regular clinical practice is hampered by other concerns. Venous reservoirs act as a safety buffer during events of massive bleeding, and as such its omission in MECC circuits may become counter-productive. The use of a cell saver requires heparinization of recruited blood and may increase postoperative blood loss. Furthermore, a switch to MECC would be limited by cost considerations; however, an improved postoperative course with MECC may demand fewer health care resources. These concerns require further study and clinical experience. An appropriate cost–utility analysis is also required to formally quantify the economic burden of MECC.

### Limitations

The main limitation of this meta-analysis lies within the methodologic variability. The CECC and MECC systems used in each paper differed significantly in circuit type, anticoagulation and cardioplegic solutions used, tube coating, and priming volumes. All these factors may impact the clinical outcomes measured, particularly regarding stroke and mortality. In addition, there were no standardized definitions for the outcomes measured between studies. The majority of studies did not explicitly state their timescale or criteria for recording mortality, arrhythmia, and stroke, and, in some, precise definitions for “neurologic events” and renal failure were not provided. To rectify this, future studies should employ standardized and clearly defined outcomes for more accurate comparisons.

The composite outcome in this study was a calculated sum of the selected end points from the extracted data. However, papers did not clearly state whether subjects had singular or multiple adverse outcomes. This composite outcome may therefore partially consist of adverse events experienced by the same subject. Also, our methods for statistical analysis did not include any correction of type 1 error which may have occurred due to the number of end points evaluated.

Several studies included in this analysis had a moderate risk of bias attributable to inadequate randomization and lack of allocation concealment. Carer blinding is not possible when providing different extracorporeal circulation techniques and is an unavoidable source of bias. However, most papers included were at low risk of bias. Although the majority of patients were undergoing CABG surgery, the rest of the studies were grouped into a non-CABG group that included minimally invasive, aortic valve, aortic root, or a combination of surgeries. Therefore, conclusions made about the non-CABG group may be confounded by the different types of surgeries performed. Future studies could assess the effects of MECC on specific non-CABG surgeries. Meta-regression of the subgroups was not performed.

This meta-analysis only included RCTs. Future meta-analyses may include cohort studies, unpublished material and ongoing trials to reduce publication bias and increase the power of the analyses, given the relatively small sample sizes for each clinical outcome assessed.

## Conclusions

This meta-analysis provides an updated comparison of MECC and CECC in postoperative outcomes. Supporting previously reported benefits, MECC demonstrated a reduced incidence of a composite of mortality, stroke, renal failure, and myocardial infarction, as well as blood loss, transfusion requirements, arrhythmias, and ICU/hospital stay. Additional studies are required to assess the long-term outcomes of MECC, using standardized definitions of endpoints such as mortality and renal failure. A cost–utility analysis is also necessary to assess the economic viability of incorporating MECC into routine clinical practice.

### Conflict of Interest Statement

The authors reported no conflicts of interest.

The *Journal* policy requires editors and reviewers to disclose conflicts of interest and to decline handling or reviewing manuscripts for which they may have a conflict of interest. The editors and reviewers of this article have no conflicts of interest.

## Figures and Tables

**Table E1:** Quality assessment of studies

Author	Year	Adequate sequence generation?	Allocation concealment used?	Blinding of participants?	Incomplete outcome data addressed?	Different outcome measurements between groups?	Free of selective outcome reporting?	Overall risk of bias
Abdel-Rahman et al^15^	2005	Yes	Yes	Unclear (PN)	Yes	No	Yes	Low
Anastasiadis et al^16^	2010	Yes	Unclear (PY)	Unclear (PN)	Unclear (PY)	Unclear (PN)	Yes	Low
Anastasiadis et al^17^	2017	Yes	Unclear (PY)	Unclear (PN)	Yes	No	Unclear (PY)	Low
Asteriou et al^18^	2013	Yes	Unclear (PY)	Unclear (NI)	Yes	No	Yes	Low
Basciani et al^19^	2016	Yes	Yes	Unclear (NI)	Yes	No	Yes	Low
Bauer et al^20^	2010	Yes	Unclear (PY)	Unclear (PY)	Yes	No	Yes	Low
Baumbach et al^21^	2016	Yes	Yes	Unclear (NI)	Yes	No	Yes	Low
Beghi et al^22^	2006	Yes	Unclear (PY)	Unclear (PY)	Yes	No	Yes	Low
Camboni et al^23^	2009	Yes	Unclear (PY)	Unclear (PN)	Yes	No	Yes	Low
Castiglioni et al^24^	2007	Yes	Yes	No	Yes	No	Yes	Low
Castiglioni et al^25^	2009	Yes	Yes	No	Yes	No	Yes	Low
Chew et al^26^	2015	Yes	Yes	Unclear (NI)	Yes	No	Yes	Low
Deininger et al^27^	2016	Yes	Unclear (PY)	Unclear (PY)	Yes	No	Yes	Low
El-Essawi et al^28^	2019	Yes	Unclear (PY)	Unclear (PY)	Yes	Unclear (PN)	Yes	Low
Elçi et al^29^	2011	Yes	Unclear (PY)	Unclear (PY)	Yes	No	Yes	Low
Farag et al^30^	2016	Yes	Unclear (PY)	Unclear (PY)	Yes	No	Yes	Low
Gunaydin et al^31^	2009	Yes	Yes	Yes	Yes	No	Yes	Low
Gygax et al^32^	2018	Yes	Unclear (PY)	Unclear (PY)	Yes	No	Yes	Low
Halfwerk et al^33^	2019	Yes	Yes	Yes	Yes	No	Yes	Low
Haneya et al^34^	2012	Yes	Unclear (PY)	Yes	Unclear (PY)	No	Yes	Low
Huybregts et al^35^	2007	Yes	Unclear (PY)	Yes	Unclear (PY)	No	Yes	Low
Kiaii et al^36^	2012	Yes	Yes	No	Yes	No	Yes	Some concerns
Kiessling et al^37^	2018	Yes	Unclear (PY)	Yes	Yes	No	Yes	Low
Kofidis et al^38^	2008	Unclear (PY)	Unclear (PY)	Unclear (PN)	Yes	No	Yes	High
Kolackova et al^39^	2012	Yes	Yes	Unclear (PN)	Yes	No	Yes	Low
Kutschka et al^40^	2009	Yes	Yes	Unclear (PN)	Yes	No	Yes	Low
Liu et al^41^	2012	Unclear (PY)	Unclear (PY)	Unclear (PN)	Yes	No	Yes	Some concerns
Modrau et al^42^	2020	Yes	Yes	No	Yes	No	Yes	Low
Nasso et al^43^	2011	Yes	Unclear (NI)	Unclear (NI)	Yes	No	Yes	High
Ng et al^44^	2015	Yes	Yes	No	Yes	No	Yes	Low
Ohata et al^45^	2008	Yes	Unclear (PY)	Unclear (PN)	Yes	No	Yes	Low
Remadi et al^46^	2004	Yes	Unclear (PY)	Unclear (PY)	Yes	No	Yes	Low
Remadi et al^47^	2006	Yes	Yes	No	Yes	No	Yes	Low
Rimpiläinen et al^48^	2011	Yes	Yes	Unclear (PN)	Yes	No	Yes	Low
Rosato et al^49^	2012	Yes	Yes	Yes	Yes	No	Yes	Low
Sakwa et al^50^	2009	Yes	No	Unclear (PN)	Yes	No	Yes	High
Schoenebeck et al^51^	2010	Unclear (PY)	Unclear (PY)	No	Yes	No	Yes	Low
Schöttler et al^52^	2008	Yes	Yes	No	Yes	No	Yes	Low
Skrabal et al^53^	2007	Yes	Yes	No	Yes	No	Yes	High
Svitek et al^54^	2009	Yes	Yes	No	Yes	No	Yes	Low
Van Boven et al^55^	2013	Yes	No	No	Yes	No	Yes	Some concerns
Yuhe et al^56^	2020	Yes	Yes	No	Yes	No	Yes	Some concerns

*PN*, Probably no; *PY*, probably yes; *NI*, no information.
